# Existence of interference between the heart and respiratory sounds: preliminary report

**DOI:** 10.1186/cc10801

**Published:** 2012-03-20

**Authors:** N Finahari

**Affiliations:** 1 Brawijaya University, Malang East Java, Indonesia

## Introduction

Heart diseases still persist as one of the first-ranked causes of mortality in the world and Indonesia. Currently, mortality from coronary heart diseases is estimated to reach 53.5 per 100,000 population [1]. Auscultation is a fundamental diagnostic method for heart disease, noninvasive and inexpensive [2], but highly dependent on the expertise and experience of the listener. Improved accuracy of diagnosis is usually then performed through further examination using the electrocardiogram, magnetic resonance imaging and the computed tomography scan. Unfortunately, these tools require very expensive investment costs that are only available in large hospitals [3]. This is the main reason for supporting the development of computer-based auscultation technique tools that are cheaper and are able to improve the accuracy and reliability of diagnosis on early stages [2]. If the device can be designed as portable, then it can be used by heart disease patients for daily monitoring to avoid or minimize heart attack accidents. To improve the accuracy of heart auscultation analysis, usually the lung sound must be minimized, or *vice versa*. It is very difficult. This study tried to use heart and lung interference sounds as physiological parameters. So this preliminary research aims to prove that interference does occur between heart and respiratory sounds. This interference sound will be used as an analysis technique to improve the accuracy of a new auscultation device.

## Methods

This research was conducted on nine randomly chosen volunteers whose heart sounds were recorded in two conditions: 30 seconds free and hold breathing. The heart sound recording process is done electronically using a modified standard stethoscope to generate digital data. Modifications were performed using a mic condenser combined with a voice processing system based on Windows XP. Accuracy of the equipment is ensured by the noise-signal ratio test.

## Results

**Figure 1:**
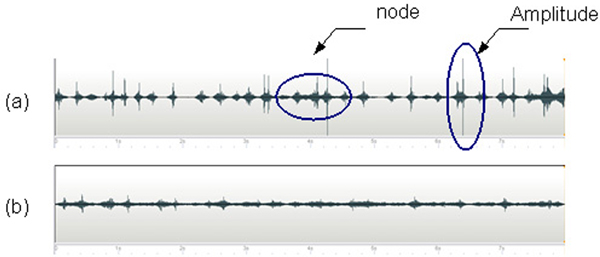
**Characteristics of the data differences between (a) free and (b) hold breathing**.

Generally, it can be seen (Figure 1) that there are pronounced differences in heart sound data recorded in the conditions of free and hold breathing. This means that the respiration process is likely to affect the heart sounds heard on the chest surface. The differences that appear are in the form of nodes and amplitude. Differences in the form of a node indicate a difference in frequency of sounds and color (timbre), while the amplitude differences may indicate differences in strength and speed of sound propagation. In general, the number of differences in the recording position is close to the number of respiratory cycles so that it is possible these differences are caused by respiratory processes.

## Conclusion

The differences that can be noticed from the graphical visualization of recorded sounds are in the form of nodes and amplitude. These differences that indicate the frequency, sound color, strength and speed of sounds improve the existence of an interference wave between the heart and respiratory sounds. These characteristics will be used to design the new portable auscultation device.
